# Continuing Immunotherapy Beyond Progression Prolongs the Survival of Patients With Extensive‐Stage Small‐Cell Lung Cancer: A Multicenter Retrospective Analysis

**DOI:** 10.1002/cam4.71607

**Published:** 2026-02-08

**Authors:** Zhuoran Sun, Yaru Tian, Shuangqing Lu, Jiling Niu, Qingfen Dong, Hui Zhu

**Affiliations:** ^1^ Department of Radiation Oncology, Shandong Cancer Hospital and Institute Shandong First Medical University and Shandong Academy of Medical Science Jinan China; ^2^ Shandong Yiyang Health Group Xinwen Central Hospital Taian China

**Keywords:** continuing immunotherapy, extensive‐stage small‐cell lung cancer (ES‐SCLC), immune checkpoint inhibitors (ICIs), immunotherapy (IO), programmed death 1 (PD‐1) inhibitor, programmed death ligand 1 (PD‐L1) inhibitor

## Abstract

**Background:**

Platinum‐based two‐agent chemotherapy combined with immunotherapy is now the first‐line standard of care for extensive‐stage small cell lung cancer (ES‐SCLC). Further studies are needed to determine whether continuing immunotherapy (IO) can provide benefit in patients whose disease has progressed after first‐line treatment. Therefore, we conducted a retrospective study to evaluate the efficacy of continuing IO in patients.

**Methods:**

The study retrospectively collected clinical data of ES‐SCLC patients as progressive disease (PD) after receiving first‐line treatment with PD‐1/PD‐L1 inhibitors. According to whether to continue immunotherapy or not, patients were divided into the continuing IO group and the control group. The differences in progression‐free survival (PFS2, defined as time from progression on first‐line treatment to progression on second‐line treatment) and overall survival (OS) between the two groups were compared.

**Result:**

As a result, a total of 489 patients from three cancer centers were enrolled in this study, of which 298 patients were included in the continuing IO group and 191 patients were included in the control group. By analysis, it was found that continuing IO could prolong OS (median: 18.82 months vs. 16.43 months, *p* = 0.008) and PFS2 (median: 4.13 months vs. 3.77 months, *p* = 0.04) compared to the control group. In subgroup analyses, continuing immunotherapy led to prolonged survival in patients with an initial efficacy evaluation of the complete response (CR) or partial response (PR). And there was also no difference in survival between the PD‐L1 inhibitor group and the PD‐1 inhibitor group in the comparison of different ICIs types.

**Conclusions:**

Continuation of immunotherapy after standard first‐line immunotherapy plus chemotherapy can improve survival in patients with ES‐SCLC.

**Abbreviations**
ChT: chemotherapy
ES‐SCLC: extensive‐stage small cell lung cancer
ICIs: immune checkpoint inhibitors
IO: immunotherapy
LS‐SCLC: limited‐stage small cell lung cancer
NSCLC: non‐small cell lung cancer
ORR: objective response rate
OS: overall survival
PD: progressive disease
PD‐1 inhibitor: programmed death 1 inhibitor
PD‐L1 inhibitor: programmed death ligand 1 inhibitor
PFS: progress‐free survival
PR: partial remission
SD: stable disease
TRT: thoracic radiation therapy

## 1 Introduction

Small cell lung cancer (SCLC) accounts for about 15% of all lung cancer cases [1, 2]. It is characterized by a high degree of malignancy, rapid tumor proliferation (Ki67 nuclear staining shows that the proliferation rate of SCLC usually reaches 70%–100%), susceptibility to distant metastasis, and extremely poor prognosis, with a usual 5‐year survival rate of < 7% [2]. SCLC can be staged according to both TNM stage and the VALG stage, and the VALG stage divides it into limited‐stage small cell lung cancer (LS‐SCLC) and extensive‐stage small cell lung cancer (ES‐SCLC), of which more than two‐thirds of patients are already in the extensive stage at the time of diagnosis. For decades, the standard of care for patients with ES‐SCLC has been four to six cycles of platinum‐based chemotherapy (ChT) combined with etoposide (EP) [3]. However, resistance to this treatment eventually develops. After disease diagnosis, systemic therapy can relieve symptoms and control disease progression, but the overall survival (OS) of ES‐SCLC patients is still short.

The addition of immune checkpoint inhibitors (ICIs) targeting PD‐1/PD‐L1 has brought benefits to patients with ES‐SCLC. PD‐L1 inhibitors including adebrelimab [4], durvalumab [5], and atezolizumab [6], and PD‐1 inhibitors including serplulimab [7], toripalimab, and tislelizumab [8] have been recommended as first‐line standard‐of‐care agents, which can result in an OS of boost of 2–3 months. Despite the benefit of immunotherapy in the first line, most patients experience progression in about 6 months. Currently, there are fewer drugs available for second‐line treatment of ES‐SCLC, and chemotherapy is still the mainstay, for example, topotecan, paclitaxel, and lurbinectin. However, the efficacy of topotecan in the treatment of recurrent SCLC is limited. Studies have shown that topotecan has an ORR of 18%, a median PFS of 2.8 months, and a median OS of 7.7 months in the treatment of sensitive recurrent SCLC, and an ORR of 2%–6% and a median OS of 6–6.5 months in the treatment of refractory recurrent SCLC [9]. Although the response rate of second‐line chemotherapy is low, mostly in the range of 10%–20%, and survival after second‐line is only 3–7 months, chemotherapy is still the best choice for these patients.

It is still unknown whether immunotherapy can be continued after disease progression, especially in patients who have had good results with first‐line immunotherapy. In studies related to non‐small cell lung cancer (NSCLC), it has been suggested that continuing immunotherapy can provide a survival benefit for patients. In a retrospective study of stage IIIB or IV NSCLC, the median survival of the continuing IO group was significantly better than that of the control group, with PFS and OS of 8.9 and 26.6 months, and a DCR of 89.7% [10]. In a retrospective study of driver‐gene negative patients with advanced NSCLC, there was a trend toward prolonged OS and PFS with continuing immunotherapy (OS: 14.1 months vs. 10.8 months, *p* = 0.063, PFS: 8.7 months vs. 4.1 months, *p* < 0.001) [11]. In another phase II BTCRC‐LUN15‐029 clinical trial results showed that patients with advanced NSCLC could significantly benefit from the use of chemotherapy combined with immunotherapy after disease progression. Both PFS and OS were prolonged compared to historical data for single‐agent chemotherapy, with a clinical benefit rate of 70.6% [12]. These studies suggested that continuing immunotherapy remains a possible benefit.

In contrast, for ES‐SCLC, there are still fewer relevant studies in this area. A study evaluating the efficacy of nivolumab in combination with temozolomide in second‐line ES‐SCLC showed an ORR of 28%, which met the prespecified criteria, a median PFS of 2.4 months, and a median OS of 6.3 months, which suggests that second‐line continuing immunotherapy is efficacious. In the IMfirst study, ES‐SCLC was treated with atezolizumab after treatment progression. Continuing immunotherapy was required to prolong survival with active treatment compared to no treatment after progression, while PFS and OS were not statistically different compared to receiving other treatments. So it remains questionable whether continuing immunotherapy can provide benefit after disease progression. In this context, we conducted this retrospective study. The aim of our study is to investigate whether continuing use of ICIs would provide a survival benefit for patients with ES‐SCLC.

## 2 Materials and Methods

### 2.1 Data Collection

The study retrospectively screened ES‐SCLC patients with disease progression after remission from first‐line treatment with ICIs between March 2019 and December 2023 at Shandong Cancer Hospital, Shanghai Chest Hospital, and Tianjin Cancer Hospital. The inclusion criteria are as follows: patients were diagnosed with ES‐SCLC at initial diagnosis; received not less than 4 cycles of chemotherapy in first‐line treatment; received PD‐1/PD‐L1 inhibitors combined with chemotherapy as first‐line treatment; confirmed disease progression after standard first‐line therapy; used systemic therapies in a second‐line regimen, with or without combination immunotherapy. The study was in accordance with the Declaration of Helsinki and the Universal Declaration of Human Rights and was approved by the Ethics Committee.

### 2.2 Treatments

In this study, patients received PD‐L1/PD‐1 inhibitors in combination with EP/EC every 21 days. Patients with stable disease after first‐line treatment received ICIs as maintenance therapy. After progression from first‐line treatment, the continuing IO group received ICIs in combination with chemotherapy or anti‐angiogenic drugs, and the control group only received chemotherapy or anti‐angiogenic drugs. Second‐line chemotherapy regimens mainly included EP/EC, paclitaxel, and topotecan. Anti‐angiogenic drugs included anlotinib and bevacizumab.

### 2.3 Assessments of Response

Tumor status is assessed after every two cycles starting with the first treatment and continuing for 4–6 cycles. The response evaluation of tumors was based on the Response Evaluation Criteria in Solid Tumors (RECIST) version 1.1.

### 2.4 Endpoint

The primary endpoint of the study was OS, and the secondary objective was PFS2. PFS1 was defined as the time from the date of diagnosis to disease progression after first‐line treatment. PFS2 was defined as the time from progression after first‐line treatment to disease progression after second‐line treatment or death. OS was defined as the time from the date of diagnosis to death or cutoff. The final follow‐up cutoff is January 15, 2025.

### 2.5 Statistical Analysis

We used the *χ* ^2^ test and Fisher exact test to compare categorical variables. Survival analysis was performed for the different groups according to the Kaplan–Meier method. Differences in survival curves between the two groups were compared using the log‐rank test, and differences were considered statistically significant when *p* ≤ 0.05, with all reported *p*‐values being two‐sided with a 95% confidence interval. Statistical analysis was performed using Prism software version 10 (GraphPad) and SPSS27.0 software.

## 3 Result

### 3.1 Patient Characteristics

**FIGURE 1:**
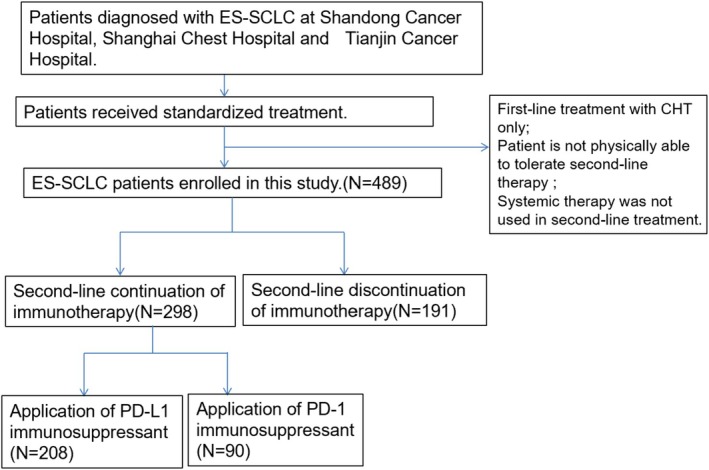
Flowchart of the screening procedure.

A total of 489 patients were enrolled in this study, 103 patients were from Shanghai Chest Hospital, 57 patients were from Tianjin Cancer Hospital, and 329 patients were from Shandong Cancer Hospital. Among them, 298 patients belonged to the continuing IO group and 191 patients belonged to the control group (Figure 1). Table 1 summarized the baseline characteristics of the patients in both groups. The median age of the total population was 62 years (ranging from 30 to 81 years). 408 (83.4%) patients were male, 312 (63.8%) had a history of smoking, and 424 patients (86.7%) had a KPS score of ≥ 80. 24.3% had brain metastases, 31.7% had liver metastases, and 36% had bone metastases. 465 patients (95.1%) received 4–6 cycles of etoposide in combination with platinum‐based chemotherapy as part of their first treatment. Prior to the first progression, patients were grouped according to their best response; 382 (78.1%) patients were evaluated as CR or PR, 107 (21.9%) as SD or PD. 143 (29.2%) patients received TRT in their first‐line therapy. With the exception of immunization cycles, most of the clinical characteristics of the patients in both groups were well balanced. 183 (61.4%) patients in the continuing IO group received ≥ 6 cycles of immunotherapy, compared with 94 (49.2%) in the control group.

**TABLE 1:** Baseline characteristics of all study patients.

Characteristics	All patients (*n* = 489) (%)	Continuing IO (*n* = 298) (%)	Control group (*n* = 191) (%)	*p*
**Age, median, years**				
< 65	294 (60.1)	173 (58.1)	121 (63.4)	0.243
≥ 65	195 (39.9)	125 (41.9)	70 (36.6)	
**Gender**				
Male	408 (83.4)	250 (83.9)	158 (82.7)	0.734
Female	81 (16.6)	48 (16.1)	33 (17.3)	
**KPS**				
≥ 80	424 (86.7)	260 (87.2)	164 (85.9)	0.66
< 80	65 (13.3)	38 (12.8)	27 (14.1)	
**Smoking status**				
Former or Current	312 (63.8)	195 (65.4)	117 (61.3)	0.348
Never	177 (36.2)	103 (34.6)	74 (38.7)	
**Brain metastasis**				
No	370 (75.7)	224 (75.2)	146 (76.4)	0.749
Yes	119 (24.3)	74 (24.8)	45 (23.6)	
**Liver metastasis**				
No	334 (68.3)	207 (69.5)	127 (66.5)	0.491
Yes	155 (31.7)	91 (30.5)	64 (33.5)	
**Bone metastasis**				
No	313 (64)	178 (59.7)	103 (53.9)	0.205
Yes	176 (36)	120 (40.3)	88 (46.1)	
**ChT cycles**				
4 ≤ *x* ≤ 6	465 (95.1)	284 (95.3)	181 (94.8)	0.788
> 6	24 (4.9)	14 (4.7)	10 (5.2)	
**IO cycles**				
≥ 6	277 (56.6)	183 (61.4)	94 (49.2)	0.008
< 6	212 (43.4)	115 (38.6)	97 (50.8)	
**Response of initial therapy**				
CR, PR	382 (78.1)	233 (78.2)	149 (78)	
SD, PD	107 (21.9)	65 (21.8)	42 (22)	0.963
**Immunization**				
PD‐1 inhibitors	137 (28)	90 (30.2)	47 (24.6)	0.179
PD‐L1 inhibitors	352 (72)	208 (69.8)	144 (75.4)	
**TRT**				
No	346 (70.8)	213 (71.5)	133 (69.6)	0.662
Yes	143 (29.2)	85 (28.5)	58 (30.4)	

Abbreviations: ChT, chemotherapy; IO, immunotherapy; KPS, Karnofsky performance status; TRT, thoracic radiation therapy.

Second‐line chemotherapy regimens mainly included EP/EC, paclitaxel, and topotecan. Anti‐angiogenic drugs included anlotinib and bevacizumab. Except for immunotherapy, there was no significant difference between the two groups in terms of second‐line systemic treatment. In the continuing IO group, 194 (65.1%) cases received immunotherapy, 32 (10.7%) cases received anti‐angiogenic therapy, and 40 (13.5%) cases received a combination of chemotherapy and anti‐angiogenic drugs. The remaining 32 (10.7%) cases were treated with immunotherapy alone. In the control group, 133 (69.6%) cases received immunotherapy, 16 (8.4%) cases received anti‐angiogenic therapy, and 42 (22%) cases received a combination of chemotherapy and anti‐angiogenic drugs (Table 2).

**TABLE 2:** Baseline characteristics of second‐line treatment.

Treatment	All patients (*n* = 489) (%)	Continuing IO (*n* = 298) (%)	Control group (*n* = 191) (%)	*p*
ChT	327 (66.9)	194 (65.1)	133 (69.6)	0.1
ChT + Anti‐angiogenesis therapy	82 (16.8)	40 (13.5)	42 (22)	
Anti‐angiogenesis therapy	48 (9.8)	32 (10.7)	16 (8.4)	
IO	32 (6.5)	32 (10.7)	0 (0)	

### 3.2 Efficacy Analysis for All Patients

**FIGURE 2:**
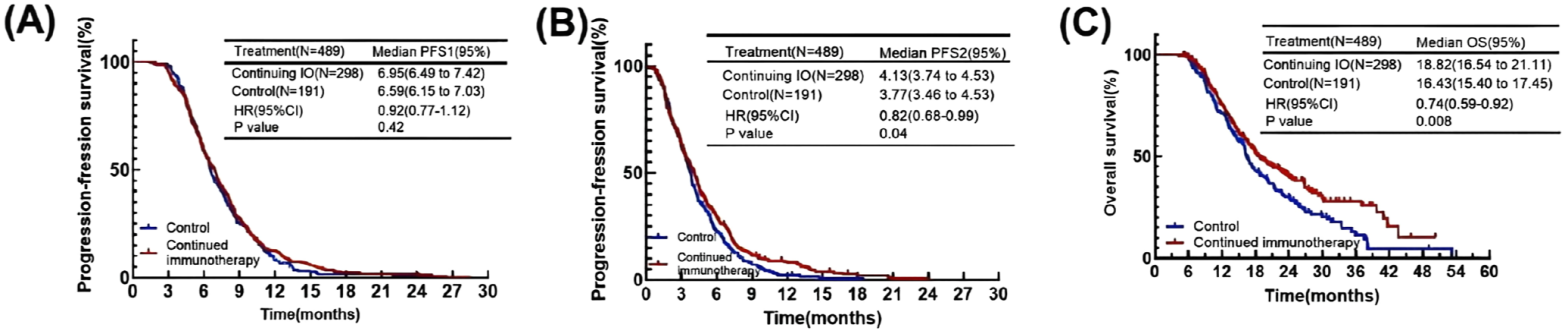
(A) Kaplan–Meier plot of PFS1 for the continuing immunotherapy group versus control. (B) Kaplan–Meier plot of PFS2 for the continuing immunotherapy group versus control. (C) Kaplan–Meier plot of OS for the continuing immunotherapy group versus control.

For all patients, the median PFS1 for first‐line treatment in the continuing IO group and the control group was 6.95 and 6.59 months, and there was no significant difference in PFS1 between the two groups (*p* = 0.42). This suggested it would not make a meaningful effect on overall survival (Figure 2A).

In the second‐line treatment, the continuing IO group showed a longer median PFS2 compared to the control group (4.13 months vs. 3.77 months, HR = 0.82, *p* = 0.04) (Figure 2B). In terms of median overall survival, the similar result could also be seen. Continuing immunotherapy beyond progression significantly prolonged the patients' survival (18.82 months vs. 16.43 months, HR = 0.74, *p* = 0.008) (Figure 2C).

### 3.3 Subgroup Analysis by Previously Received Efficacy Assessment

We also analyzed patients in groups based on the efficacy of first‐line treatment. Based on efficacy, patients were categorized into CR, PR group (382 cases) and SD, PD group (107 cases). The best remission was 382 patients in the CR, PR group, of which 233 were in the continuing IO group and 149 were in the control group. Of the 107 patients in the SD, PD group, the cases of patients in each of the two groups were 65 and 42.

**FIGURE 3:**
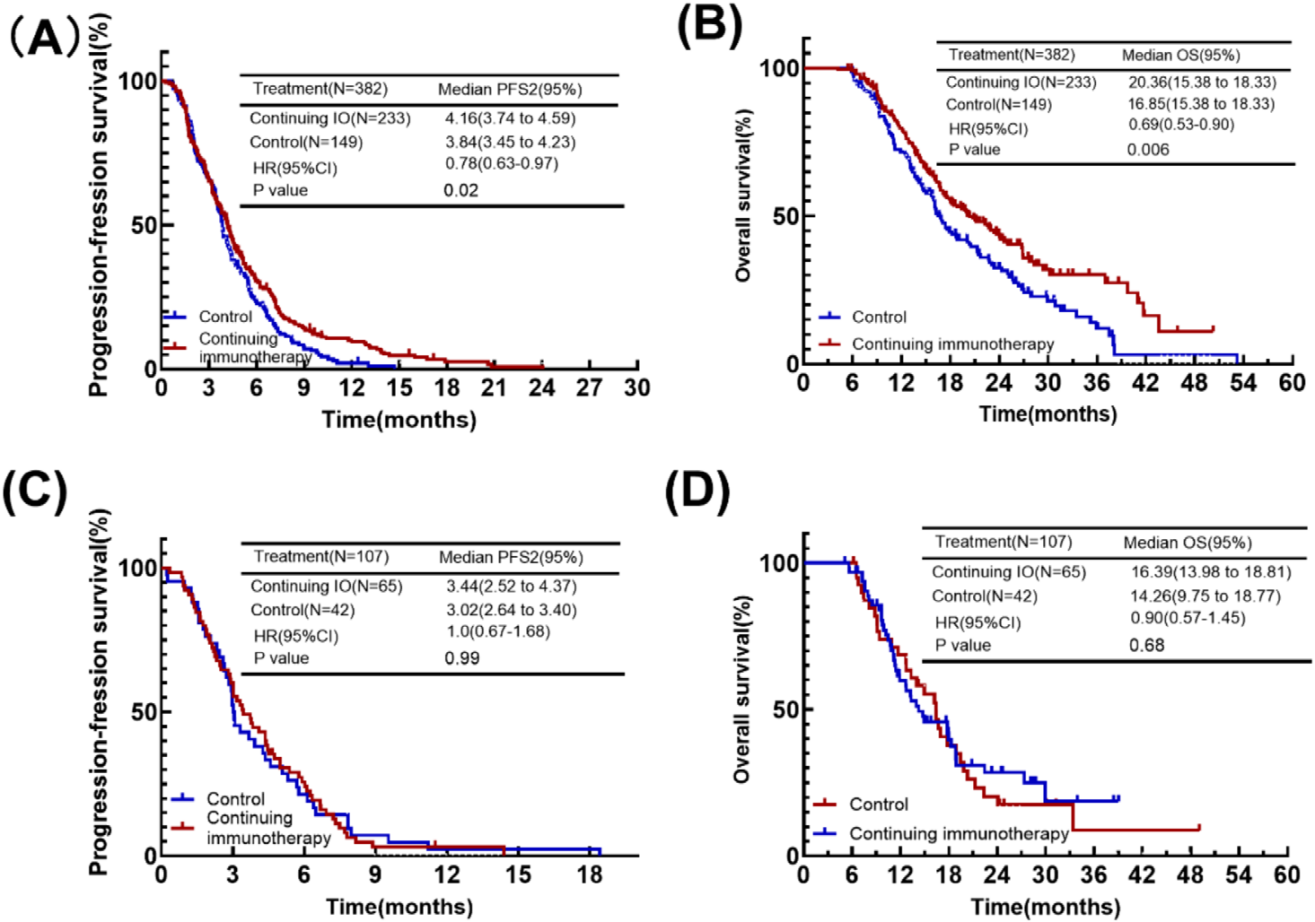
(A) Kaplan–Meier plot of PFS2 for the continuing immunotherapy group versus control group in the CR, PR group. (B) Kaplan–Meier plot of OS for the continuing immunotherapy group versus control group in the CR, PR group. (C) Kaplan–Meier plot of PFS2 for the continuing immunotherapy group versus control group in the SD, PD group. (D) Kaplan–Meier plot of OS for the continuing immunotherapy group versus control group in the SD, PD group.

First we analyzed whether the response to continuing immunotherapy was different by efficacy at first‐line treatment. In patients evaluated for CR or PR, those in the continuing IO group showed significant survival benefits, including PFS2 and OS (mPFS2: 4.16 months vs. 3.84 months, HR = 0.78, *p* = 0.02; mOS: 20.36 months vs. 16.85 months, HR = 0.69, *p* = 0.006) (Figure 3A,B). But in those evaluated for SD or PD, patients in the continuing IO group had a longer mPFS2 and mOS (mPFS2: 3.44 months vs. 3.02 months; mOS: 16.39 months vs. 14.26 months). However, the difference between the two groups was not statistically significant (PFS: *p* = 0.99; OS: *p* = 0.68) (Figure 3C,D). Therefore, patients who achieved better efficacy were more likely to benefit from continuing immunotherapy.

### 3.4 Subgroup Analysis by Second‐Line Treatment

In the second‐line treatment, we divided the patients into groups based on their treatment options. In addition to immunotherapy, systemic treatments for both groups were chemotherapy, chemotherapy combined with anti‐angiogenesis therapy, and anti‐angiogenesis therapy. So we analyzed whether continuing immunotherapy in the presence of different second‐line treatment regimens would be beneficial in all cases.

**FIGURE 4:**
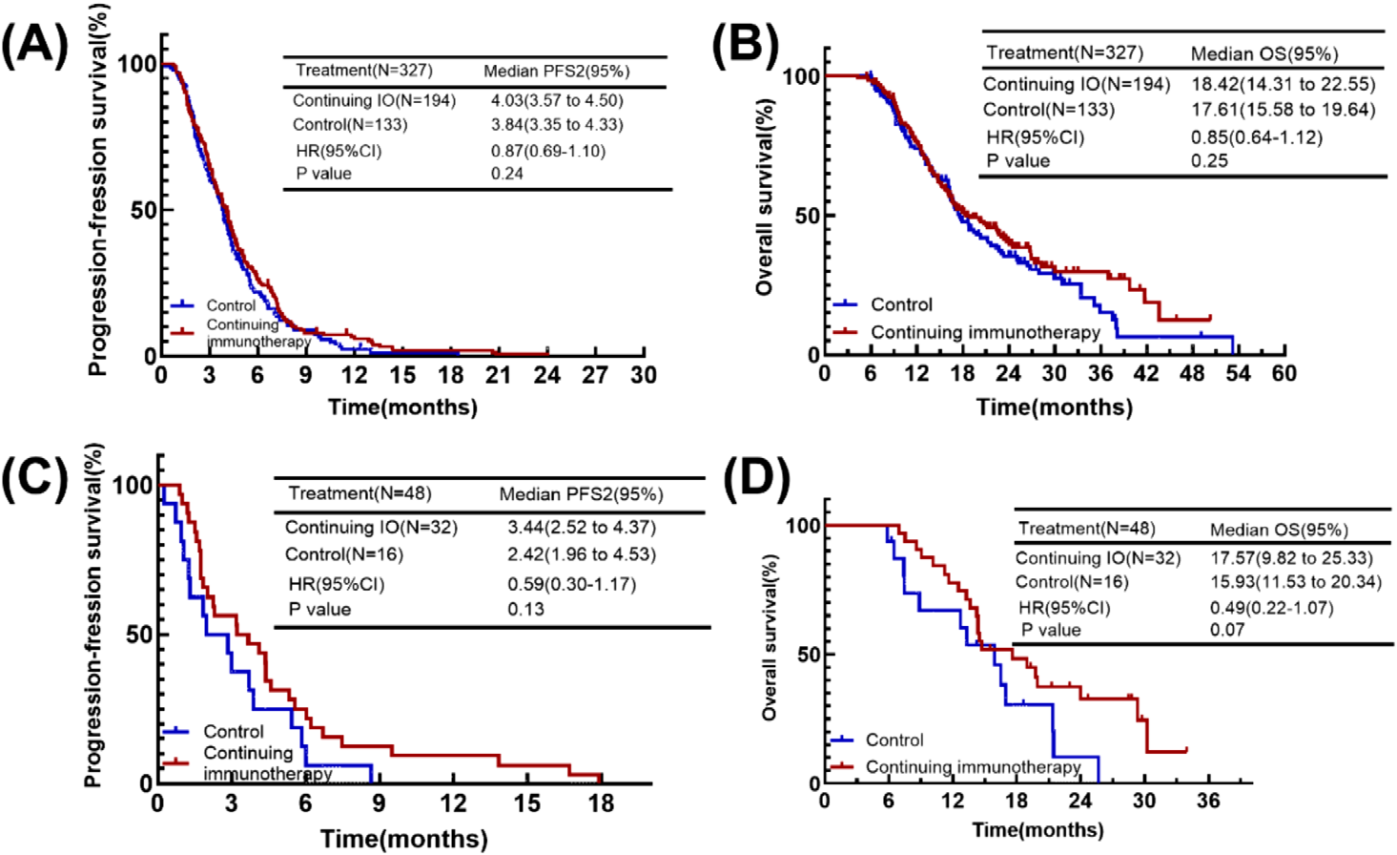
(A) Kaplan–Meier plot of PFS2 for the continuing and control groups with only using chemotherapy in second‐line treatment. (B) Kaplan–Meier plot of OS for the continuing and control groups with only using chemotherapy in second‐line treatment. (C) Kaplan–Meier plot of PFS2 for the continuing and control groups with only using anti‐angiogenesis therapy in second‐line treatment. (D) Kaplan–Meier plot of OS for the continuing and control groups with only using anti‐angiogenesis therapy in second‐line treatment.

In the patients received chemotherapy, although there was a trend toward prolonging patients PFS2 and OS, continuing immunotherapy did not result in a statistically significant difference (mPFS2: 4.03 months vs. 3.84 months, *p* = 0.24; mOS: 18.42 months vs. 17.61 months, *p* = 0.25) (Figure 4A,B). In patients received anti‐angiogenesis therapy, there was also a trend toward prolonging PFS2 and OS (mPFS2: 3.44 months vs. 2.42 months, *p* = 0.13; mOS: 17.57 months vs. 15.93 months, *p* = 0.07) (Figure 4C,D). Despite the fact that we did not see statistically significant differences in these two comparisons, continuing immunotherapy has a tendency to prolong patient survival regardless of the second‐line treatment options.

### 3.5 Subgroup Analysis by Different Baseline Metastases

**FIGURE 5:**
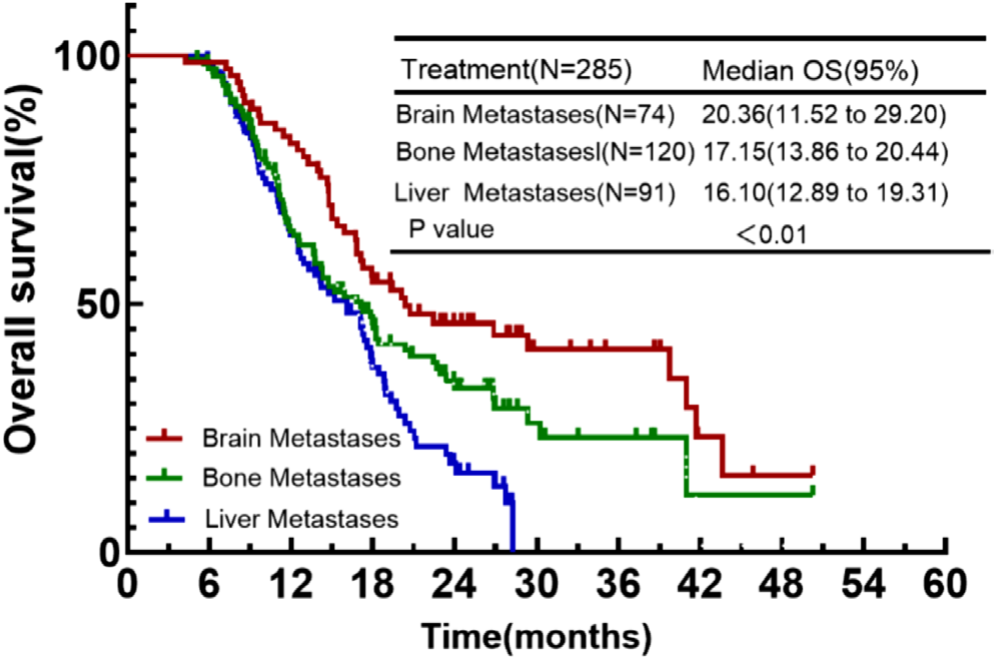
Kaplan–Meier plot of OS for the presence of baseline metastases at initial diagnosis.

The initial diagnosis of ES‐SCLC in patients was usually accompanied by metastases to distant organs. And different metastatic organs have different effects on prognosis. In the continuing IO group, 74 (24.8%) cases had brain metastases, 120 (40.3%) cases had bone metastases, and 91 (30.5%) cases had liver metastases. In the control group, 45 (23.6%) cases had brain metastases, 88 (46.1%) cases had bone metastases, and 64 (33.5%) cases had liver metastases. In the continuing IO group, those with brain metastases were more likely to benefit from continuing immunotherapy, followed by patients with bone metastases, while those with liver metastases benefited only to a limited extent (mOS: 20.36 months vs. 17.15 months vs. 16.10 months) (Figure 5).

**FIGURE 6:**
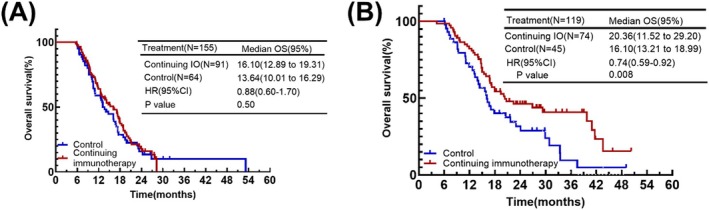
(A) Kaplan–Meier plot of OS for the presence of liver metastases at initial diagnosis. (B) Kaplan–Meier plot of OS for the presence of brain metastases at initial diagnosis.

In patients with liver metastases, continuing immunotherapy did not prolong patient survival (mOS: 16.10 months vs. 13.64 months, HR = 0.99, *p* = 0.50) (Figure 6A). However, in patients with brain metastases, continuing immunotherapy demonstrated a clear advantage (mOS: 20.36 months vs. 16.10 months, HR = 0.74, *p* = 0.008) (Figure 6B).

### 3.6 Subgroup Analysis by Type of ICIs

**FIGURE 7:**
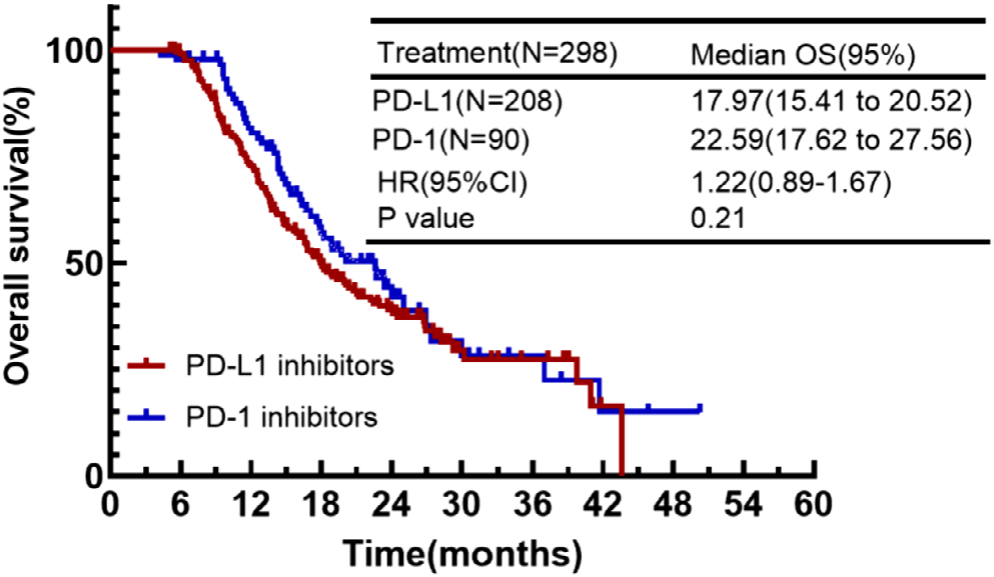
Kaplan–Meier plot of OS with PD‐L1 inhibitors applied to therapy versus PD‐1 inhibitors.

Of the 298 patients in the continuing IO group, 208 used programmed death ligand 1 (PD‐L1) inhibitors and 90 used programmed death 1 (PD‐1) inhibitors. The comparative analysis of the two groups showed that the type of ICIs did not have a significant effect on the OS of the patients (mOS: 17.97 months vs. 22.59 months, HR = 1.22, *p* = 0.21) (Figure 7).

### 3.7 Subgroup Analysis by Whether to Add Thoracic Radiation Therapy

**FIGURE 8:**
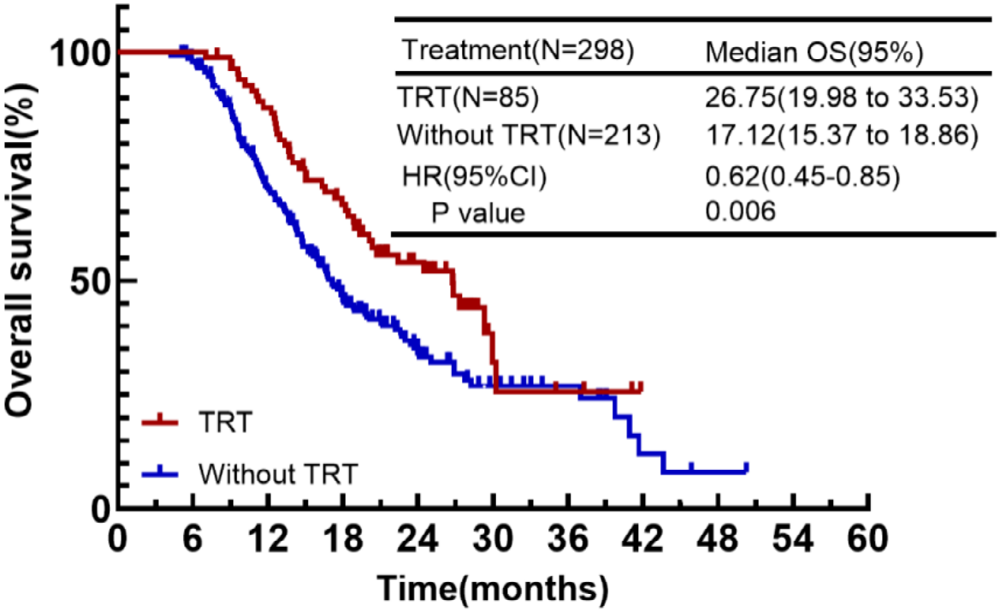
Kaplan–Meier plot of OS for the effect of TRT on efficacy.

In first‐line treatment, in addition to systemic therapy, some patients also received thoracic radiation therapy. In the continuing IO group, 85 cases (28.5%) added TRT while 213 cases (71.5%) did not. We further analyzed and found that the OS of patients who received TRT was significantly prolonged and significantly superior to that of patients who did not receive TRT (mOS: 26.75 months vs. 17.12 months, HR = 0.62, *p* = 0.006) (Figure 8).

### 3.8 Prognostic Factors

The clinical characteristics of the patients were evaluated to determine their prognostic value for OS (Table 3). Univariate analysis showed that age, gender, baseline metastasis, cycles of first‐line immunotherapy, and response of initial therapy were associated with survival. Multivariate analysis indicated an effect of age and liver metastases on survival. Patients < 65 (*p* < 0.01) and without liver metastasis (*p* < 0.01) were favorable prognostic factors for OS.

**TABLE 3:** Univariate and multivariate analysis of OS.

Factors	Univaruate analysis of OS HR (95% CI)	*p*	Multivariate analysis of OS HR (95% CI)	*p*
Age (< 65/≥ 65)	1.52 (1.22–1.91)	< 0.01	1.38 (1.10–1.73)	< 0.01
Gender (male/female)	0.71 (0.52–0.97)	0.03	0.81 (0.56–1.16)	0.25
KPS (≥ 80/< 80)	0.99 (0.72–1.38)	0.97		0.84
Smoke (smoking/never)	0.89 (0.71–1.12)	0.33		0.75
Brain (no/yes)	1.28 (0.99–1.66)	0.07		0.49
Liver (no/yes)	0.51 (0.41–0.64)	< 0.01	0.56 (0.44–0.71)	< 0.01
Bone (no/yes)	0.72 (0.58–0.91)	< 0.01	0.83 (0.66–1.05)	0.11
ChT (4–6/< 4; > 6)	1.03 (0.63–0.96)	0.89		0.16
IO (< 6/≥ 6)	0.77 (0.61–0.96)	0.02	1.24 (0.98–1.55)	0.07
Response of initial therapy (CR + PR/SD + PD)	1.37 (1.05–1.78)	0.02	1.24 (0.95–1.63)	0.11
ICIs (PD‐1/PD‐L1)	0.82 (0.64–1.05)	0.11		0.35

### 3.9 Toxicities

We counted the incidence of adverse reactions for both first‐ and second‐line treatments. The incidence of any grade of toxicity [3] (e.g., hematologic toxicity, hepatic and renal impairment, and gastrointestinal dysfunction) as well as grade ≥ 3 adverse reactions did not differ significantly between the two groups. In addition, patients who continued to receive second‐line immunotherapy did not develop severe immune‐related pneumonia, myocardial injury, or abnormal thyroid function. No patients died from drug toxicity during treatment (Table 4).

**TABLE 4:** Treatment of adverse events.

	All patients (*N* = 489)		Continuing IO (*N* = 298)		Control group (*N* = 191)		*p*
**Adverse events**	Any grade, %	Grade ≥ 3, %	Any grade, %	Grade ≥ 3, %	Any grade, %	Grade ≥ 3, %	
**Hematologic toxicities**							
Leukocytopenia	261 (53.2)	74 (15.1)	157 (52.7)	43 (14.4)	104 (54.5)	31 (16.2)	0.75
Neutropenia	232 (47.5)	80 (16.4)	144 (48.3)	49 (16.4)	88 (46.1)	31 (16.2)	0.9
Anemia	411 (84.2)	21 (4.3)	245 (82.2)	16 (5.4)	166 (86.9)	5 (2.6)	0.13
Thrombocytopenia	183 (37.4)	24 (5.0)	124 (41.6)	13 (4.4)	59 (30.9)	11 (5.8)	0.19
Lymphocytopenia	390 (79.9)	102 (20.9)	245 (82.2)	62 (20.8)	145 (75.9)	40 (20.9)	0.71
**Gastrointestinal disorder**							
Nausea or vomiting	260 (53.2)	10 (2.0)	157 (52.7)	6 (2.0)	103 (53.9)	4 (2.1)	0.98
Diarrhea	24 (5.0)	0 (0)	13 (4.4)	0 (0)	11 (5.6)	0 (0)	0.51
Constipation	102 (20.9)	0 (0)	70 (23.5)	0 (0)	32 (16.8)	0 (0)	0.15
**Hepatic function damage**							
Transaminase increased	14 (2.9)	3 (0.6)	10 (3.3)	0 (0)	4 (2.1)	3 (1.6)	0.24
Creatinine increased	4 (0.8)	0 (0)	1 (0.3)	0 (0)	3 (1.6)	0 (0)	0.14
**Immunotherapy related pneumonia**	61 (12.5)	0 (0)	42 (14.1)	0 (0)	19 (9.9)	0 (0)	0.23
**Imunotherapy related heart injury**	45 (9.2)	0 (0)	29 (9.7)	0 (0)	16 (8.4)	0 (0)	0.64
**Imunotherapy related thyroid dysfunction**	46 (9.4)	0 (0)	29 (9.7)	0 (0)	17 (8.9)	0 (0)	0.78

## 4 Discussion

The addition of immunotherapy has changed the situation of ES‐SCLC. However, after first‐line treatment, most patients quickly develop resistance and relapse. In other solid tumors such as melanoma and NSCLC, several studies have affirmed the efficacy of immune cross‐line therapy. However, there are fewer relevant studies in ES‐SCLC and the findings are inconsistent. In the IMfirst study, patients evaluated for PD after first‐line immunotherapy in combination with chemotherapy or immune maintenance therapy were divided into three groups: continuing IO, chemotherapy, and no treatment. The mOS of the three groups were 14.3, 12.6, and 7.2 months, respectively, with a statistically significant shorter OS in the no‐treatment group, demonstrating that aggressive therapeutic measures should be taken after progression to prolong survival. However, there was no significant difference between the continuing IO and chemotherapy groups. In a Chinese real‐world study, 63.63% of patients who progressed on first‐line immunotherapy received immune cross‐line therapy. The PFS1 + PFS2 was longer than the control group, but did not reach the significance threshold (median PFS 1 + PFS2 11.27 months vs. 7.20 months, *p* = 0.19) [13]. In another retrospective analysis, the study included a total of 100 patients with ES‐SCLC that progressed with immunotherapy. After balancing the clinical baseline characteristics using IPSW (Inverse Propensity Score Weighted), the continuing IO group showed significant improvement in PFS and OS (mPFS: 4.5 vs. 2.8 months, *p* = 0.017; mOS: 11.6 vs. 5.4 months, *p* = 0.028) [14]. Overall, the role of continuing IO in ES‐SCLC patients has not been fully elucidated.

Immunotherapy, unlike other treatments, kills cancer cells indirectly by either unsuppressing the immune cells or activating the immune cells themselves. In the process of tumor immune response, T cells are the most important effector cells in fighting against tumors, while immunosuppressants can activate T cells and restore the killing effect on tumor cells [15, 16]. Once immunotherapy takes effect, it lasts a long time. This is mainly due to the memory function of the immune cells, which will continue to kill or inhibit tumor cells, also known as the “trailing effect”. Therefore, long‐term immunotherapy can continuously activate immune cells, increase immune reserve, and effectively prolong the survival of patients. This was also reconfirmed in our study; continuing immunotherapy could prolong both PFS2 and OS.

And further analyzing the population that might benefit, we found that patients who had better outcomes on first‐line therapy were more likely to benefit from continuing immunotherapy, in patients with best response of CR or PR, PFS2 and OS were significantly longer in the continuing IO group than in the control group. However, this difference was not seen in patients with SD or PD. We hypothesized that this could be related to the heterogeneity of the tumor. Because even for the same tumor, patients have different immune microenvironments and molecular typing. In order to better parse the SCLC population that could precisely benefit from immunotherapy, Prof. Byers utilized RNA‐seq from patients in the Impower133 clinical trial for related exploration. The study revealed the presence of four subtypes, SCLC‐A, SCLC‐N, SCLC‐P, and SCLC‐I [17, 18]. Subsequent prognostic analyses suggested that the SCLC‐I subtype population could significantly benefit from atezolizumab+ chemotherapy treatment compared to the placebo+ chemotherapy group population, with a median OS of 18.2 versus 10.4 months in both groups, respectively. Compared to the overall population's 2‐month median OS improvement, the SCLC‐I subtype gained a 7.8‐month median OS improvement, the SCLC‐P subtype gained a 3.6‐month median OS improvement, whereas no significant median OS improvement was observed for SCLC‐A, SCLC‐N. It can be seen that different subtype of SCLC can have a very significant impact on treatment response and prognosis. However, SCLC typing has not been popularized in the clinic yet, and the road to achieve precise treatment is still full of difficulties.

Some ES‐SCLC patients have brain metastases and liver metastases at the time of initial diagnosis. Previously this was seen as a poor prognostic factor. With the advent of the immunization era, the prognosis of different metastatic regions has been reassessed. For a long time, the brain has had limited efficacy of systemic therapy due to the blood–brain barrier [19]. However, recent studies have shown that T cells can cross the blood–brain barrier. Immunotherapy, on the other hand, by activating the immune system and enhancing T‐cell activity, can successfully enter the intracranial area to inhibit tumor growth [5, 6, 20, 22]. The results of our subgroup analysis also reaffirmed that in patients with brain metastases, continuing immunotherapy is effective in prolonging patient survival (mOS: 20.36 months vs. 16.10 months, *p* = 0.008). Liver metastases, however, have a diminished efficacy of immunotherapy due to its specific immune environment [22]. The liver is immunotolerant, and Kupffer cells in it can induce the expansion and activation of CD4+ regulatory T cells to promote systemic tolerance, as well as express Fas ligand (FasL) and eliminate activated T cells entering the liver. And several trials have also demonstrated that while the addition of immunotherapy in either NSCLC or SCLC can prolong patient survival to a certain extent, the benefit is less compared to patients without liver metastases [22, 23, 24]. Our study showed that continuing IO cannot provide further benefit to the patients with liver metastases.

In second‐line treatment, patients in both groups received systemic regimens, including chemotherapy and anti‐angiogenesis therapy. When the disease progresses after previous immunotherapy or immune maintenance therapy, the addition of a new systemic regimen might reduce the body's resistance to immunologic drugs. According to the choice of second‐line treatment regimens, this study analyzed whether different drugs combined with immunotherapy could all benefit. As could be seen by the results, the addition of immunotherapy, either in combination with chemotherapy or anti‐angiogenesis therapy, resulted in prolonged OS. Relatively speaking, the efficacy of anti‐angiogenesis therapy in combination therapy was superior to that of chemotherapy. And patients using anti‐angiogenesis therapy in the second line were more likely to benefit from combination immunotherapy, especially PFS2. In a prospective clinical trial, sintilimab was combined with anlotinib for ES‐SCLC in the treatment of second‐line and above, with a final median PFS of 6.1 months; the primary endpoint was successfully met [25]. In the combined effect, anti‐angiogenesis therapy exerts its anti‐cancer effect by inhibiting tumor angiogenesis. It can also improve the vascular structure of tumor tissues, increase the infiltration of immune cells in tumor tissues, and reduce the drug resistance of immunotherapy, thus enhancing the efficacy of immunotherapy.

The immunologic drugs commonly used in ES‐SCLC patients are categorized as PD‐1 and PD‐L1 inhibitors. Some trials have shown that PD‐1 inhibitors are more efficacious than PD‐L1, and both have comparable safety profiles [26]. In the subgroup analysis, we also compared patients with two different inhibitors. The results showed that patients with PD‐1 inhibitors had better median OS than those with PD‐L1, but the difference between the two was not significant. If a more accurate comparison of the two effects on survival is desired, prospective studies are worthy to confirm this.

In the era of immunotherapy, increasing evidence suggests that the addition of radiotherapy can improve antitumor efficacy. The synergistic mechanism between the two mainly focuses on how radiation therapy can reshape the immune microenvironment and release cytokines and inflammatory factors. Meanwhile, immunotherapy can improve tumor hypoxia and increase radiation sensitivity. However, there are still some opinions that adding radiotherapy increases the incidence of adverse reactions, such as pneumonia. In a phase II trial, the addition of TRT following chemotherapy combined with immunotherapy significantly improved patient survival. OS for patients receiving TRT and those not receiving TRT was 22.9 and 13.4 months. PFS also showed a significant difference, at 11.3 and 4.1 months, respectively [27]. Our study also confirmed this finding: patients in the continuing IO group who received TRT had better OS than those who did not receive (mOS: 26.75 months vs. 17.12 months). And in terms of adverse events, continuing immunotherapy necessarily increased the risk of immunotherapy‐related pneumonia. However, the overall adverse events were comparable to those in the control group, and the incidence of immune‐related injury was within acceptable limits.

In summary, how existing treatment methods can maximize the survival period of ES‐SCLC patients remains a question that needs to be explored. Immunotherapy has now become one of the first‐line treatment regimens for ES‐SCLC, but in clinical practice, patients often continue immunotherapy in second‐line treatment due to various factors. This article aims to explore whether continuing immunotherapy can bring survival benefits to patients. It provides references for patients in choosing different types of ICIs, which patients are more likely to benefit from continuing immunotherapy, and whether patients with different baseline metastases can obtain the same benefits. It can bring more choices to patients' second‐line treatment options.

Inevitably, there are a number of limitations to our study. First, although we balanced, as far as possible, the differences in the main clinical baseline characteristics between the groups, there were other potential factors that we did not take into account, which affected the results of the study. In addition, the study only provided a rough categorization of second‐line treatments, did not include other treatments such as radiotherapy, and did not differentiate and assess the efficacy of each treatment regimen specifically. Finally, retrospective studies have their inherent shortcomings, namely recall bias and selection bias. Therefore, we need a large‐scale, multicenter, prospective study to further validate this.

## 5 Conclusion

Our study demonstrated that continuation of immunotherapy after progression on first‐line standard therapy for ES‐SCLC could prolong PFS2 and OS in patients with ES‐SCLC. In addition, some patients who had better outcomes in first‐line therapy, or who had brain metastases, can benefit more from continuing immunotherapy. This study focused on the analysis of second‐line immunotherapy for ES‐SCLC patients in the IO era, and our findings may provide additional treatment options for ES‐SCLC patients after disease progression.

## Author Contributions

**Zhuoran Sun:** conceptualization (equal), data curation (equal). **Yaru Tian:** data curation (equal), formal analysis (equal). **Shuangqing Lu:** formal analysis (equal), investigation (equal), methodology (equal). **Jiling Niu:** investigation (equal), methodology (equal). **Qingfen Dong:** project administration (equal). **Hui Zhu:** project administration (equal).

## Funding

This work was supported by the National Natural Science Foundation of China (82103632) and the Natural Science Foundation of Shandong (ZR2021QH245).

## Ethics Statement

This study was approved by the Institutional Review Board of Shandong Cancer Hospital and Institute and was performed in accordance with the Declaration of Helsinki.

## Consent

This pilot study is an exception to the rule, being a retrospective study, not using human samples, and posing no risk to subjects. Based on the above, the Institutional Review Board of Shandong Cancer Hospital and Institute agreed to waive the informed consent of the subjects.

## Conflicts of Interest

The authors declare no conflicts of interest.

## Data Availability

All the data generated or analyzed during this study are included in this published article. The datasets used and/or analyzed during the current study are available from the corresponding authors.
